# Linking Human Health and Livestock Health: A “One-Health” Platform for Integrated Analysis of Human Health, Livestock Health, and Economic Welfare in Livestock Dependent Communities

**DOI:** 10.1371/journal.pone.0120761

**Published:** 2015-03-23

**Authors:** S. M. Thumbi, M. Kariuki Njenga, Thomas L. Marsh, Susan Noh, Elkanah Otiang, Peninah Munyua, Linus Ochieng, Eric Ogola, Jonathan Yoder, Allan Audi, Joel M. Montgomery, Godfrey Bigogo, Robert F. Breiman, Guy H. Palmer, Terry F. McElwain

**Affiliations:** 1 Paul G. Allen School for Global Animal Health, Washington State University, Pullman, Washington, United States of America; 2 Center for Global Health Research, Kenya Medical Research Institute, Kisumu, Kenya; 3 Centers for Disease Control and Prevention, Nairobi, Kenya; 4 School of Economic Sciences, Washington State University, Pullman, Washington, United States of America; 5 Animal Diseases Research Unit, Agricultural Research Service, United States Department of Agriculture, Pullman, Washington, United States of America; 6 Emory University, Atlanta, Georgia, United States of America; New York City Department of Health and Mental Hygiene, UNITED STATES

## Abstract

**Background:**

For most rural households in sub-Saharan Africa, healthy livestock play a key role in averting the burden associated with zoonotic diseases, and in meeting household nutritional and socio-economic needs. However, there is limited understanding of the complex nutritional, socio-economic, and zoonotic pathways that link livestock health to human health and welfare. Here we describe a platform for integrated human health, animal health and economic welfare analysis designed to address this challenge. We provide baseline epidemiological data on disease syndromes in humans and the animals they keep, and provide examples of relationships between human health, animal health and household socio-economic status.

**Method:**

We designed a study to obtain syndromic disease data in animals along with economic and behavioral information for 1500 rural households in Western Kenya already participating in a human syndromic disease surveillance study. Data collection started in February 2013, and each household is visited bi-weekly and data on four human syndromes (fever, jaundice, diarrhea and respiratory illness) and nine animal syndromes (death, respiratory, reproductive, musculoskeletal, nervous, urogenital, digestive, udder disorders, and skin disorders in cattle, sheep, goats and chickens) are collected. Additionally, data from a comprehensive socio-economic survey is collected every 3 months in each of the study households.

**Findings:**

Data from the first year of study showed 93% of the households owned at least one form of livestock (55%, 19%, 41% and 88% own cattle, sheep, goats and chickens respectively). Digestive disorders, mainly diarrhea episodes, were the most common syndromes observed in cattle, goats and sheep, accounting for 56% of all livestock syndromes, followed by respiratory illnesses (18%). In humans, respiratory illnesses accounted for 54% of all illnesses reported, followed by acute febrile illnesses (40%) and diarrhea illnesses (5%). While controlling for household size, the incidence of human illness increased 1.31-fold for every 10 cases of animal illness or death observed (95% CI 1.16–1.49). Access and utilization of animal source foods such as milk and eggs were positively associated with the number of cattle and chickens owned by the household. Additionally, health care seeking was correlated with household incomes and wealth, which were in turn correlated with livestock herd size.

**Conclusion:**

This study platform provides a unique longitudinal dataset that allows for the determination and quantification of linkages between human and animal health, including the impact of healthy animals on human disease averted, malnutrition, household educational attainment, and income levels.

## Introduction

In livestock-dependent households and communities, the health of humans, livestock, and household economic welfare are closely linked. It is estimated nearly one billion people living on less than 2 dollars a day, 300 million of whom reside in sub-Saharan Africa, depend on livestock as a source of livelihood and nutrition [1]. These figures represent two thirds of the rural poor and one third of the urban poor whose food, income, social status, and store of wealth depend on livestock [2,3]. However, the relationship between livestock health and productivity, and human health and welfare is complex, and understanding it in quantitative terms remains a critical requirement for developing sustainable poverty relief and public health interventions through human and livestock health maintenance and improvement.

The linkages between livestock keeping and human nutrition and health outcomes have been conceptualized[2,4]. Broadly, they include positive effects of keeping livestock that generally improve a household’s health and welfare status; and negative effects of livestock ownership that may worsen human health and nutritional status. The positive effects include increased access to nutritious animal source foods (ASFs), such as milk, meat, and eggs, in households owning animals, and higher household cash incomes that increase purchase power for ASF’s, food crops, healthcare and education. The consumption of ASF’s provides high-quality protein, essential structural fats, and highly bioavailable essential micronutrients (e.g. zinc, iron, calcium, vitamin A, vitamin B-12) that are strongly associated with improved growth, health and cognitive ability of children[5–8], and increased resistance to and recovery from infectious diseases[9,10]. All of these have multiplier effects at the community level through better nourished children becoming more intelligent, healthier and more productive adults[2].

The negative effects associated with livestock keeping may include risk of transmission of zoonotic pathogens from animals to humans e.g. anthrax, leptospirosis, trypanosomiasis, rabies,—many of which are neglected[11], food-borne diseases e.g. cysticercosis, taeniosis, cryptosporidiosis and brucellosis, development of antimicrobial resistance, and chronic diseases such as cardiovascular disease, cancers, and diabetes associated with excessive consumption of the energy-dense high level saturated ASF’s. For instance, the rural communities in Western Kenya where this study is based suffer concurrent high levels of poverty[12,13] and high burden of infectious diseases[14], including zoonotic ones such as Q-fever, cysticercosis, cryptosporidium and trypanosomiasis[15–19].

The goal in developing policies aimed at reducing poverty is to maximize the positive linkages of livestock keeping while minimizing the negative effects, especially for people living in poverty and vulnerable groups including pregnant women and children. Quantifying the impact of livestock diseases on human health and welfare, identifying priority diseases including new zoonoses to target for greatest gain amidst limited resources, identifying the factors that enhance or reduce nutritional outcomes, and the use of this information to guide program and policy formulation for improved human health outcomes remain a major challenge[20,21].

Herein we describe a platform for integrated health and economic welfare analysis designed to address this challenge by simultaneously collecting data from a population-based animal syndromic surveillance (PBASS) system and an ongoing human population-based infectious disease surveillance (PBIDS) system in the same rural households in Western Kenya[14], and linking these data to a quantitative assessment of socio-economic dynamics in the same households over time. We provide baseline epidemiological data on the animal and human disease syndromes under investigation, and descriptive data on household demographics, wealth and assets, livestock ownership, household food consumption and expenditures, and investments in health care. Using data from the first year of study, we examine potential linkages between animal and human health and economic welfare and present these, as examples of how the data can be utilized to better understand the complex relationships that impact the rural poor.

## Materials and Methods

### Ethical review statement

Ethical clearance for the population based animal syndrome surveillance (PBASS) study was obtained from the Kenya Medical Research Institute Ethical Review Committee and Animal Care and Use Committee (reference number SSC Protocol no. 2250). The population based infectious diseases study (PBIDS) received approval from the Kenya Medical Research Institute Ethical Review Committee (reference SSC Protocol no. 1899) and the Centers for Disease Control and Prevention. Participants over 18 years of age provided a written consent to participate in these studies, and parental consent was provided for children involved in the study.

### Study population

The PBASS study is conducted in Western Kenya within a Health and Demographic Surveillance System (HDSS), which started in September 2001, and is run by the Kenya Medical Research Institute (KEMRI) in collaboration with the Centers for Disease Control and Prevention, Kenya (CDC). The HDSS provides general demographic and health information including population age-structure, in and out migrations, fertility rates, birth and death rates, verbal autopsy, access and utilization of health care for a population of approximately 220,000 individuals, in 54,869 households[22,23]. The HDSS area covers 385 villages that lie to the North-East of Lake Victoria covering Rarieda, Siaya and Gem Districts in Siaya County, an area predominantly inhabited by the Luo ethnic group practicing subsistence farming and fishing. The overall population density for the area is estimated at 325 persons per square kilometre.

Within the HDSS is the Population-Based Infectious Disease Surveillance (PBIDS) platform, which monitors the health of approximately 24,000 persons living in 6,000 households in 33 villages. The PBIDS study was initiated in 2005 to define the burden of the major infectious disease syndromes, describe epidemiologic patterns of disease, identify etiology of disease, and implement and evaluate the health impact of interventions in the study population[14,24]. Data on four disease syndromes (fever, jaundice, diarrhea and respiratory illness) for each household resident are collected through bi-weekly home visits conducted by community interviewers (CIs) in the local language. The CIs have secondary school certificate education, with additional training on the protocol, protection of human subjects, conducting a limited set of physical examinations and administering standardized questions about recent symptoms among household members.

**Fig 1:**
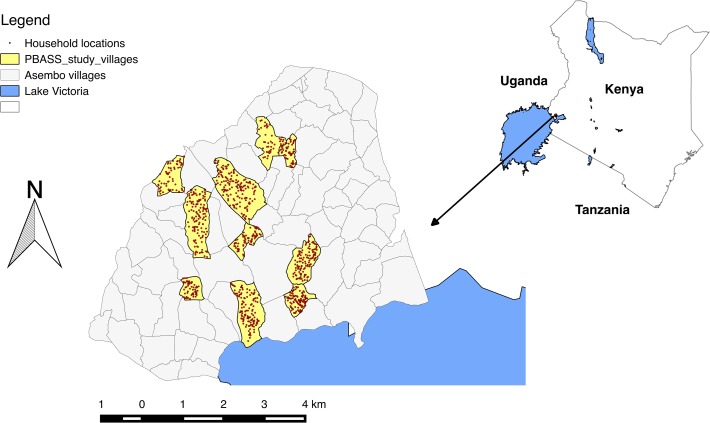
Map of the study area showing the study villages (in yellow) and the location of households (black dots) enrolled in the study.

The PBASS study is conducted in 1500 households in 10 villages randomly selected from the 33 villages participating in the PBIDS study (Fig. 1). To be included in the PBASS sample frame, households must be participating in the PBIDS study and provide written informed consent for participation in the PBASS study. All households within the 10 villages and participating in the PBIDS study are eligible for enrolment into PBASS.

Once enrolled into the PBASS study, each household is followed longitudinally, and three groups of data collected: animal disease syndrome data, human disease syndrome data, and socio-economic data for each study household.

#### Animal disease syndrome data.

The PBASS study monitors the health of cattle, goats, sheep and chickens and collects bi-weekly data on 9 syndromes: respiratory system disorders, gastro-intestinal system disorders, nervous system disorders, reproductive system disorders, digestive system disorders, urogenital system disorders, skin disorders, udder disorders, and death. A summary of syndromes and the key signs associated with each syndrome are provided in Table 1.

**Table 1:** A summary of the syndromes in cattle, goats, sheep and chickens investigated in the PBASS study.

Syndromes	Clinical signs
Reproductive disorders	abortions, stillbirths, neonatal deaths
Respiratory disorders	cough, nasal discharge, difficulty breathing
Digestive disorders	diarrhea, bloating, inappetence
Urogenital disorders	vaginal discharges, preputial discharges, scrotal swelling
Musculo-skeletal disorders	lameness, recumbency
Skin disorders	hair loss, lumps, itching
Nervous disorders	circling, incoordination
Udder disorders	mastitis (unusual colour or consistency of milk), drop in milk yield
Death	

Animal disease syndrome data are collected in two ways: 1) through community interviewers that visit each study household bi-weekly and administer a questionnaire designed to identify if any of the cattle, sheep, goats or chickens in the household have been sick with any of the listed syndromes or have died in the 2 week period preceding the interview, and 2) through a toll-free telephone number provided to all households in the study (>70% of households have access to a mobile phone), allowing the farmers to report cases of animal illness or death directly to the PBASS study team without having to wait for the next bi-weekly visit.

Following alerts of animal illness or death coming through either the CI or the toll-free number, a PBASS animal health team consisting of veterinarians and animal health assistants responds by visiting the affected household within 24 hours. The animal health team conducts a complete clinical examination of each sick animal and collects samples including blood smears, serum samples, milk samples, faecal samples, skin scrapings and any other clinically relevant samples. In cases of death, a full post-mortem examination is conducted. In addition, the team administers a detailed questionnaire that captures data on herd health including herd demographics, breeding history, and specific syndromes/clinical signs observed in the study animals. In order to aid clinical diagnosis for veterinary interventions, a limited set of diagnostics mainly involving microscopy on blood and lymph node smears obtained from sick animals are conducted. Samples collected during these visits are shipped to the KEMRI laboratory for storage for future analysis. A comprehensive diagnostic workup to establish an etiologic diagnosis is not currently conducted but will be carried out in the future to establish aetiologies for the syndromes with the greatest socio-economic impact on households, and to identify zoonotic pathogens that may cause illness in humans.

#### Human disease syndrome data.

In the same households monitored for illnesses and death in animals, human morbidity data is collected bi-weekly, simultaneously with the animal health data. These data are collected as part of the PBIDS study. Briefly, a questionnaire investigating whether any member of household has had any of the 4 syndromes (fever, jaundice, pneumonia/cough, or diarrhoea) in the 2-weeks preceding the current visit is administered. Fever is defined as axillary temperature ≥ 38.0°C, and diarrhoea as ≥3 looser than normal stools in a 24-hour period. In addition, data on whether any household member who fell ill sought health care, type of care sought, and where health care was sought from is collected. Study participants reporting any of the listed syndromes are referred (or can self-refer) to the St. Elizabeth Mission Hospital in Lwak (located within a 5km radius of study households), where free medical care is provided.

The simultaneous collection of animal health and human health data from the same households enables the linking of the two datasets and evaluation of the relationships between human and animal health.

#### Socio-economic survey data.

A detailed socio-economic survey is carried out every 3 months in each PBASS study household. The three-month return interval represents a balance between survey cost and information loss; however household socioeconomic characteristics either do not tend to change at the same temporal scale as health indicators, and because respondent recall can be (and are) relied on to capture household activity and socio-economic changes between surveys. A team of community interviewers specially trained to conduct household socio-economic surveys collects these data. Table 2 provides a summary of the type of socio-economic data collected.

**Table 2:** Description of the household socio-economic data collected every 3 months.

Group of variables	List of variables
Household demographics	Household identifier; household size; ages of household members, gender, education level, occupation
Household assets	Number and value of farm implements, electronics, bicycles, bikes and motor-vehicles owned; type of housing, absence/presence of a latrine, electricity, type of water source, amount of time taken to fetch water and number of trips per day, source of fuel, person and household member involved in firewood collection, income for each member of the household, savings accounts and balance, loans borrowed, amount and purpose of loan
Household consumption	For each food; milk (from cows, sheep and goats), meat, eggs, fish, maize, cassava, sorghum, banana, pulses, green vegetables, potatoes—amount produced at home, purchased, consumed, and the costs for each food. List of foods fed to children less than 5 years. Home expenses on fuel, clothing, health, education, and any other expense. Records on vaccinations, medication, consultation, transport, and other health costs.
Land and crop inventory	Size of land owned and/or rented, land rent costs, crop losses and value, cost of inputs (seeds, fertilizers, manure, labor) for maize, potato, sorghum, cassava, groundnuts, beans; acreage covered by each crop, size of harvest, quantity sold and sale value for each of the crops, purchases of any of the crops or gifts received
Livestock ownership	Number of calves, heifers, bullocks, cows, bulls, goats, sheep, poultry and donkeys owned on or off farm
Livestock Inventory	Number of calves, heifers, bullocks, cows, bulls, goats, sheep, poultry, donkeys born, dead, sold, consumed, received as gift, given as gifts, purchased or lost since previous visit, livestock value lost
Livestock income	Sale value for livestock sold (cattle, sheep, goats, sheep, donkeys, poultry), amount and sale value of cow, goat and sheep produced, number of eggs produced, number and sale value of eggs sold, any other income from livestock
Livestock expenditure	Purchase costs for cattle, goats, sheep, donkeys, poultry; labor costs, costs for vector (e.g. ticks and tsetse flies) control, helminth control, treatment costs, veterinarian costs, and any other costs for cattle, sheep, goats, donkeys and poultry
Animal health care	Method of tick control, distance covered by cattle for dipping/spraying, frequency of tick control per month, total cost of tick control, vaccinations in animals
Human illness	Sickness among any household members, sum of number of work days missed due to illness, number of health clinic/hospital visits made in the last 3 months, travel time to clinic/hospital in hours, records of vaccination of children under 5

**Fig 2:**
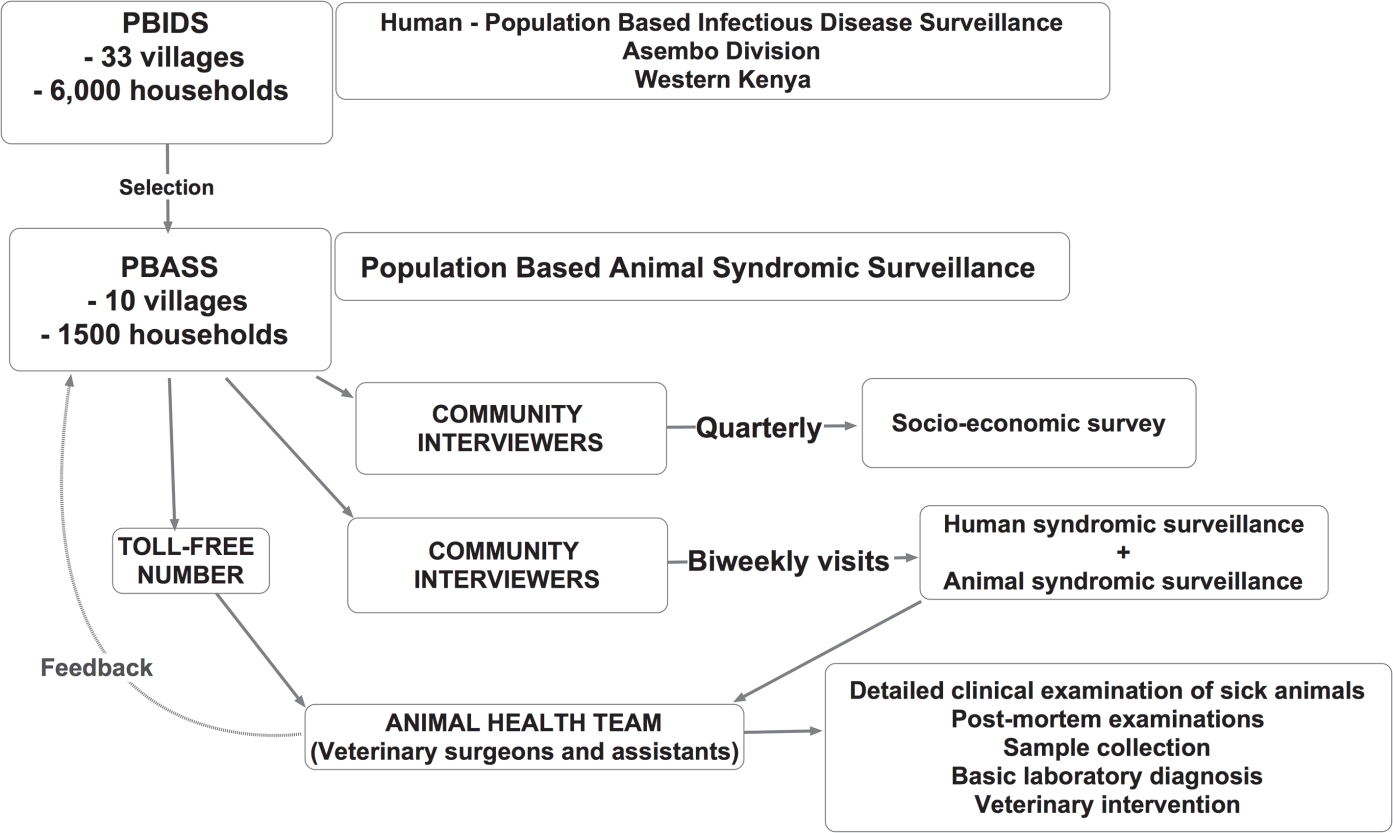
Schematic diagram showing the design of the population based animal syndromic surveillance (PBASS) Study. All PBASS study households participate in the population based infectious disease surveillance (PBIDS) study, thereby generating human health, animal health, and socio-economic data linkable at the household level.

The PBASS study design is summarised in a schematic diagram in Fig. 2.

### Data analysis

Data collection for the PBASS study began in February 2013, and a total of 1500 households provided informed consent to participate in the study. The data on human health, animal health and socio-economic status used in this analysis was collected between February 2013 and February 2014. Pocket digital assistants were used to record the data in the field, which was later downloaded and stored in a Microsoft Access database. Descriptive analyses were conducted to provide baseline numbers for variables on household demographics, animal ownership, and indicators of socio-economic status.

A household illness index for humans and for animals was computed by determining the total number of cases of illnesses in humans, and illnesses and deaths in animals per household observed in first one year of the study. The human illness index was treated as the main outcome variable. To account for the over-dispersion in the household illness index data, negative binomial regression methods were used to estimate the relationship between household illness index and household demographics, socio-economic indicators and illnesses and deaths observed in animals. All data analyses were done using the R platform for statistical computing[25].

## Results

### Household characteristics

**Fig 3:**
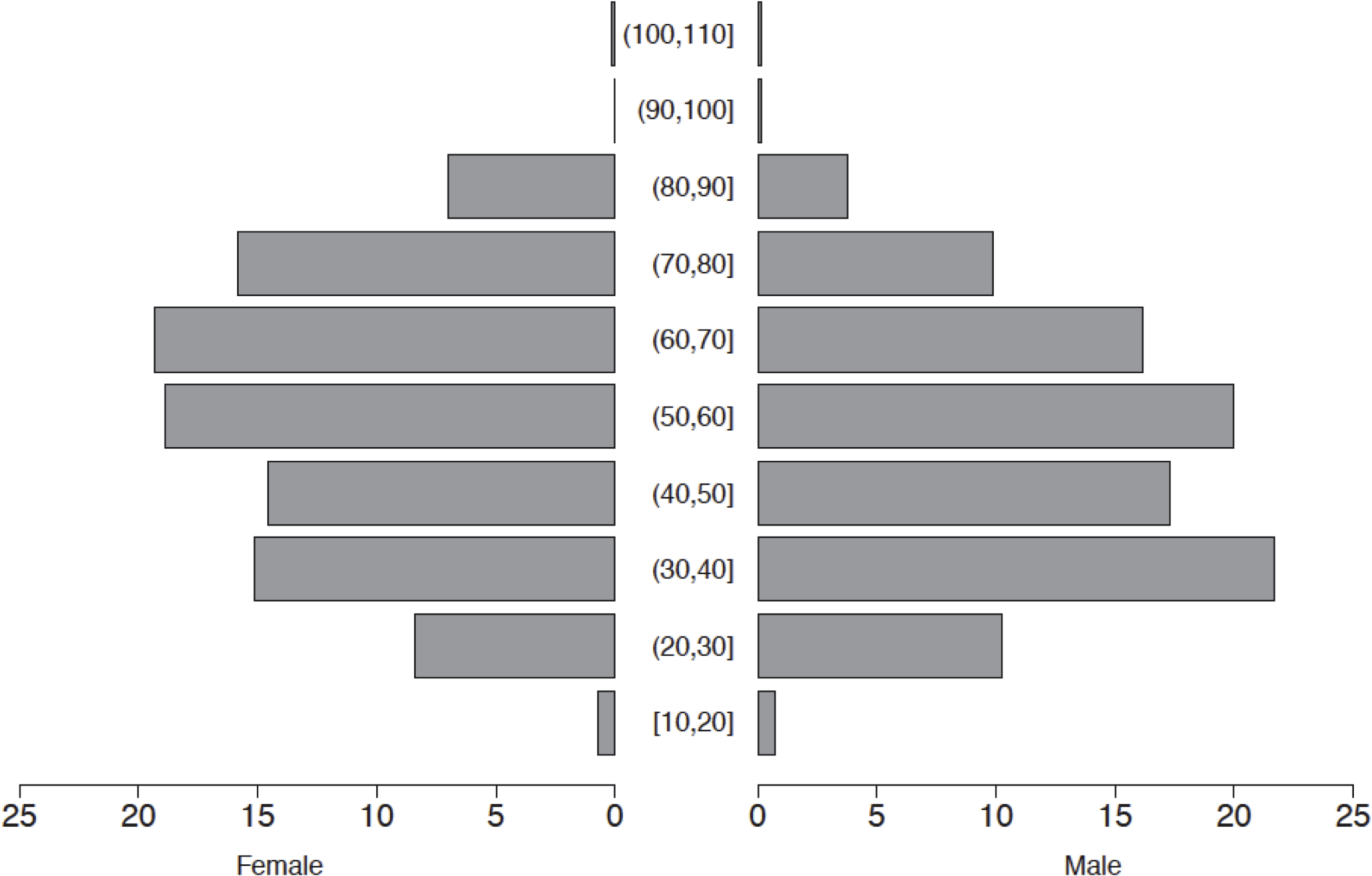
Population pyramid showing the age structure of the household heads by gender in years. The x-axis represents a percentage of each age group by gender.

The total population of people in the PBASS study households was 6,400, with an average family of 4 persons per household. The mean age of the household heads was 53 years, and the distribution of the ages of household heads is shown in Fig. 3. Thirty-three percent of the households had at least one child < 3 years, 42% had at least one child < 5 years, and 56% had one or more children < 10 years old. Up to 85% of the household heads had received some formal education. Most households (80%) were dependent on agriculture for their livelihood, and mainly practiced mixed crop-livestock production system. A summary of the household demographics is provided in S1 Table.

Data collected on the ownership of household assets (see S2 Table) shows the mean household asset value (consisting of assets such as farm implements, electronics, bicycles) to be USD 100 (Kshs. 8,820) with a median household asset value of USD 50 (Kshs. 4,200). Farming was the main source of household income, with 32% of the households reporting to receive some income away from their farms. For households that reported an off-farm income, the monthly average was USD 280 (Kshs. 23,840) and a median of USD 124 (Kshs. 10,500).

Firewood is the main source of cooking energy for 94% of the households, 5% used charcoal, and 1% used natural gas or paraffin. Among the households using firewood, only 3% reported regularly buying firewood, with 97% reporting to collect it from the surrounding environment. Electricity connection in the study area was minimal with only 3% of households being connected to the electricity grid. Most households (82%) have at least one outdoor latrine, while the rest (18%) reported having no latrine available within their homestead.

Forty-three percent of the households obtained water for domestic use from wells, 32% harvested rainwater or accessed it from seasonal streams, while the rest fetched their water from communal reservoirs or from Lake Victoria. Sixty percent of the households spent 1–2 hours each day collecting water, 20% spent under one hour a day, and 20% over 2 hours each day.

Only 7% of the enrolled households do not keep livestock. Of the 93% that keep at least one species of livestock, chickens were the most common species (88%), followed by cattle (55%), goats (41%) and sheep (19%), see Table 3. Most households practice mixed small-scale farming with a median of 9 ruminants (cattle, goats and/or sheep) and 11 adult chickens.

**Table 3:** Proportion of households keeping each species of livestock, and the summary statistics for number of animals per household.

Livestock ownership	Frequency (%)	Mean	Median	Maximum
Own least 1 species	1348 (93.4)			
Cattle	800 (55.4)	5	3	98
Goats	587 (40.7)	4	3	30
Sheep	268 (18.6)	5	3	47
Chicken	1272 (88.1)	14	11	100

Ninety-one percent of the households reported growing maize in the planting season preceding the recruitment visit. Details of the other crops grown are provided in Table 4. The sources of animal proteins for the households include fish (88%), cow milk (75%), beef (33%), and eggs (29%), Table 4. Household food consumption for the 7 days preceding the recruitment visit, the type of food consumed and the proportion of families consuming different food types are also presented in Table 4.

**Table 4:** Showing proportion of households planting each of the common crops in the most recent planting season, and foods consumed in the last 7 days preceding the interview.

Crops planted	Frequency (%)
Maize	1312 (91)
Potato	116 (8)
Sorghum	68 (5)
Cassava	61 (4)
Beans	501 (35)
Household consumption	
Cow milk	1085 (75)
Goat milk	3 (0.2)
Eggs	416 (29)
Beef	476 (33)
Goat meat	7 (0.5)
Fish	1275 (88)
Cassava	514 (36)
Sorghum	458 (32)
Banana	210 (15)
Pulses	661 (46)
Onions, carrots	1316 (91)
Green vegetables	1365 (95)
Sweet potato	366 (25)
Irish potato	105 (7)

To determine if a household’s likelihood of consuming animal source foods is associated with livestock ownership, we tested the relationship between milk and egg consumption with the total number of cows and chickens owned, respectively. The likelihood of milk consumption in a household increased by 62% for every increase by one in the number of cows owned, and the likelihood of egg consumption by 28% for every increase in the number of chickens owned (in 10 chicken units) (Table 5).

**Table 5:** Results of regression between household human illness cases and animal illness and death cases (while accounting for household size), relationship between seeking health care and livestock ownership, household income and assets and livestock ownership, and household animal source food consumption and livestock ownership.

Variable	Estimate	Lower CI	Upper CI	*p-*value
Household illness index				
(Animal illness and death/10)	1.31	1.16	1.49	< 0.001
Household size	1.08	1.07	1.1	< 0.001
By syndromes				
Gastro-intestinal tract illness (humans)				
Animal—Gastro-intestinal illness	1.06	1.02	1.12	0.009
Respiratory syndromes				
Animal—respiratory syndromes	1.1	1.04	1.17	0.002
By socio-economic status				
Household illness index				
Household head education level- formal	1.02	0.85	1.21	0.248
Number of cattle owned	1	0.99	1.01	0.241
Household consumption				
Cow milk consumption (yes/no)				
Number of cows owned	1.62	1.44	1.86	< 0.001
Egg consumption (yes/no)				
Number of Chicken owned (10)	1.28	1.17	1.4	< 0.001
Expenditure on health (yes/no)				
Number of cattle owned	1	0.97	1.02	0.906
Number of Chicken owned (10)	1.18	1.09	1.3	< 0.001
Household asset value				
Number of cattle owned	1.1	1.07	1.13	< 0.001

Over half of the households (54%) did not regularly control for ticks in their livestock and only 2% of the households reported vaccinating their cattle against any diseases. For the period of 3 months preceding the recruitment visit, 52% of the respondents reported expenditures on health care for household members (median expenditure of 2 US dollars per month per household). We tested whether the probability of expenditure on human health care was related with livestock ownership. Using the recruitment data, we found no correlation relationship between probabilities of a household spending on health during the last 3 months with the total number of cows, goats or sheep, but there was a correlation with the number of chickens owned [incidence rate ratio 1.18, 95%CI (1.09, 1.3) for every 10 chickens owned].

### Livestock disease syndromes

Whereas data for all 9 syndromes were collected in cattle, sheep, and goats, household visits for cases in chickens were made only when the mortality was >30% of the flock. Between February 2013 and February 2014, a total of 2193 cases of livestock illness and deaths were recorded. Of these, 75% were illnesses in cattle, sheep and goats and 25% were mortality cases in cattle, sheep, goats, and chicken. Cattle contributed 58% of all cases of illness, with goats and sheep contributing 28% and 14% of the cases respectively. Most of the recorded deaths (54%) occurred in chickens and chicks (only considered if >30% of the flock died), with cattle, goats and sheep contributing 14%, 22% and 10% respectively. Interestingly, the two methods for reporting illnesses or deaths in animals contributed nearly equally. The use of a toll-free number with farmers contacting the PBASS team directly to report cases of illness or deaths accounted for 46% of all the reports, while the bi-weekly visits conducted by the community interviewers accounted for 54% of all reports.

**Fig 4:**
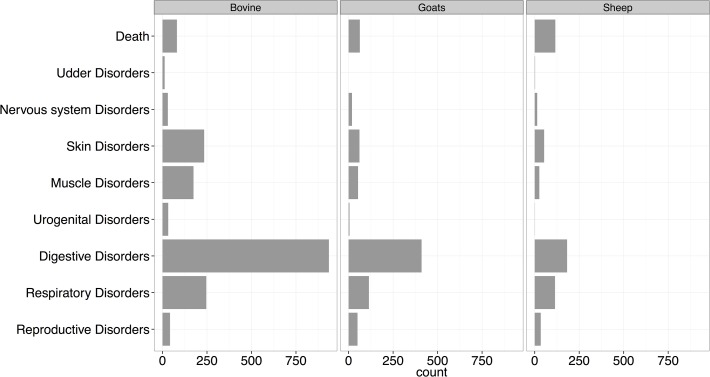
Distribution of the disease syndromes by species for data collected over the first 12 months of the study (February 2013—February 2014).

Of the illness cases reported among each livestock species, gastro-intestinal tract syndrome cases were the most common, comprising of 52%, 53%, and 34% of total cases in cattle, goats, and sheep respectively. For these three species, respiratory syndromes were the second most common observed, see Fig. 4.

### Human disease syndromes

**Fig 5:**
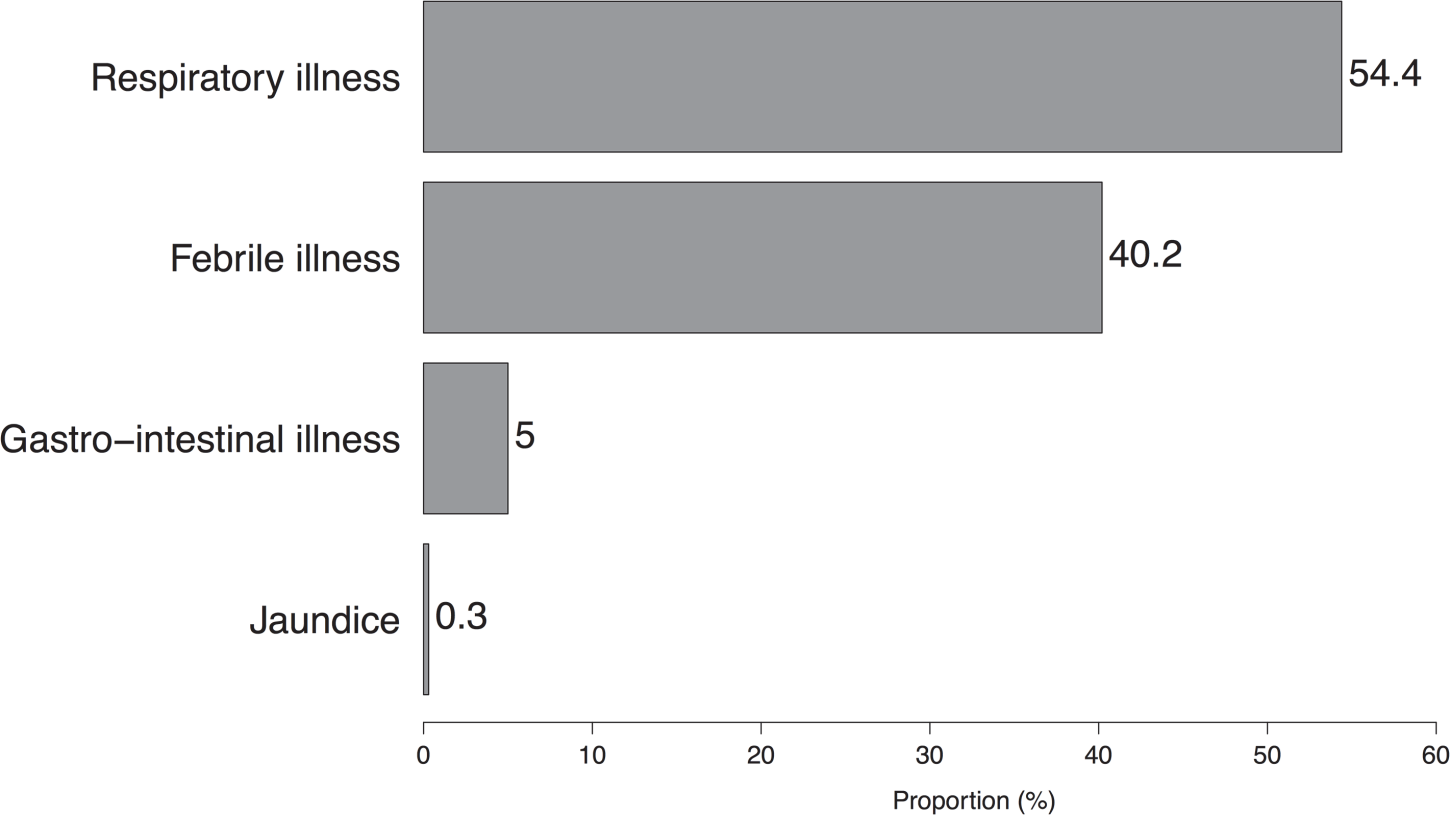
Showing a summary of the distribution of the 4 human syndromes under investigation.

Between February 2013 and February 2014 a total of 38,208 human illness cases were reported, with respiratory illnesses constituting 54.4%, see Fig. 5.

**Fig 6:**
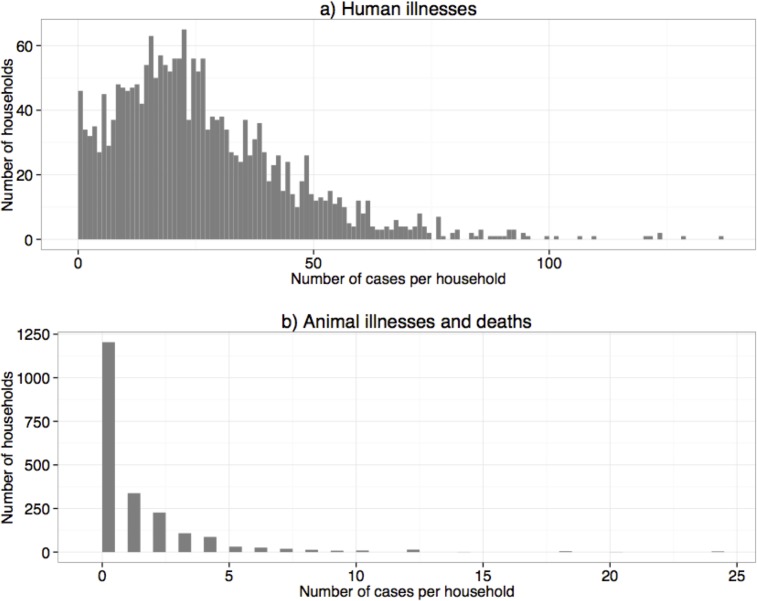
Distribution of the number of human and animal illnesses and deaths per household during the first 12 months of the study.

Using the human and animal health data collected over the 12-month period, we tested whether there was a correlation between households reporting illnesses among household members, and those reporting cases of illnesses or death in animals. To do this, we computed a summary statistic for each household, which provided the total number of illnesses reported per household in humans, and in animals over the one-year observation time. The mean number of cases of human illness per household was 26 (median 13, minimum 0, maximum 136), with a mean of 1.2 cases among livestock (median 0, minimum 0, maximum 24). An individual (human or animal) can provide as many cases to the household aggregate as were recorded through the routine surveillance visits. The distribution of these human and animal cases per household is provided in Fig. 6.

Using a negative binomial generalised linear model to account for the over-dispersed nature of the data, we tested the hypothesis that there is no correlation between human illness and animal illness cases at the household level, based on the one-year surveillance data collected. The outcome variable for this analysis was the total number of human illnesses per household, and this was regressed against the number of animal illness and deaths cases in the same households. While accounting for the household size, the results suggested that for every 10 cases of animal illness or death observed in a household, the odds of observing a human illness in the same household was 31% greater (95% CI, 16–49%).

When this analysis was extended to determine if there was an association between gastro-intestinal syndromes and respiratory syndromes in humans and animals, we found that there was a 6% (95% CI, 2–12) increase in the probability of gastro-intestinal illness in humans associated with each additional case of gastro-intestinal illness observed in animals in the same households. A 10% (95% CI, 4–17) increase in the probability of respiratory illness case in humans was associated with each additional case of respiratory illness observed in animals (Table 5).

There was no evidence of an association between the household illness index and socio-economic factors such as number of cattle owned and education level of the household head. However, the likelihood of health care seeking among household members falling ill increased with the number of cattle owned by 1.15 times (95% CI 1.03, 1.29) for every 10 cattle owned, and with the net off-farm income by 1.03 times (95% CI 1.01, 1.06) for an increase in income of 100 US dollars. Additionally, we found the asset value of a household was positively correlated to the number of cattle owned (see Table 5).

## Discussion

The PBASS study platform is designed to investigate and understand the links between human health, animal health, and specific socio-economic variables. The key strength of this study is the simultaneous collection of longitudinal data on human health, animal health, and socio-economics from the same households over time. This “One-Health” approach that allows for an integrated human-animal health and economic framework for assessing causes of poor human health have been previously proposed[26–28].

The study presumes that human health and animal health may be linked through 3 main pathways: i) a socio-economic pathway where improved livestock production is associated with healthy livestock leading to improved household incomes and wealth, access to education and health care, ii) a nutritional pathway where owning healthy livestock increases access to animal source foods that in turn reduces the risk of malnutrition and disease, and iii) a zoonotic pathway where healthier livestock are less likely to transmit zoonotic and food-borne infections. Our data is linkable at the household level, making it possible to identify the pathways by which human and animal health are related, determine the magnitude and direction of effect of each pathway, and test interventions in the animal sector that would increase human opportunity, including human health, wealth and welfare.

Although the results we present are based on analysis of only the first 12 months of data, we find strong associations between the cumulative number of human illnesses in a household (human illness index) and the cumulative number of animal illnesses and death (animal illness index) in the same households. Additionally, for the common respiratory and gastro-intestinal illnesses, households with high numbers of animal cases of each of these syndromes have high numbers of the similar syndromes in humans. We hypothesize that the observed relationships between households with high numbers of human illnesses and those with high numbers of animal illnesses may be driven by either the zoonotic pathway, with pathogens shared between humans and the animals they keep, or driven by factors that increase a household’s illness index. In the latter case, illnesses may be related to the environment in which the humans and animals co-reside. Disease clustering is known to be associated with environments that are suitable for survival of parasites and the vectors that transmit them, and where disparity conditions promote disease[29]. Although the role of environment and environmental factors on human and animal illness is not investigated in this current study, this will be a subject of analysis moving forward. Human and animal illnesses may be mediated through human and animal health management, nutrition, and healthcare-seeking activities, which in turn are mediated by wealth, income, as well as credit and liquidity constraints. This concept of social and economic determinants of health is well recognized, with greater likelihood of poor health associated with people of lower socioeconomic status[30].

We find a correlation between a household’s asset accumulation and health seeking behaviour, and the number of animals owned. Families owning large herds of cattle had assets of a greater value compared to those owning fewer animals. It is possible that asset accumulation is a result of increased income from livestock, or asset accumulation and livestock accumulation are independent but influenced by a third source of income. Such dynamics will be investigated using the multi-year data available from this longitudinal study. Asset accumulation has been related to increased income from livestock[31]. In relation to health, the likelihood of health seeking following human illness increased with number of cattle owned. These examples demonstrate the potential pathways that may relate livestock ownership to human income and wealth, and access to health care.

The hypotheses that human illnesses are driven by zoonotic infections will be tested by determining the etiologies of the human syndromes and animal syndromes observed, and the risk factors associated with specific infectious diseases identified. Currently, only limited diagnostic work is being performed to determine the etiology of the observed animal disease syndromes, until the syndromes associated with the highest economic impact at the household can be determined.

The collection of human health and animal health data at the household level is done at least every 2 weeks. Although the 2-week inter-visit interval may be associated with some recall decay[24], the interval is optimal in balancing the risk of excessive recall bias associated with longer intervals between consecutive household visits, the risk of participant fatigue with more frequent visits, and the demand on resources required for surveillance through home visits. The use of a toll-free number as an extra tool for surveillance in livestock allows the farmer to reach the veterinary team whenever an animal falls sick. This surveillance tool allows for the capture of active clinical cases, and reduces the risk of under-estimating the incidence of illnesses associated with recall bias. From this study, approximately half of all recorded cases of illness and death in animals were reported through the toll-free number, a relatively inexpensive, and efficient alternative surveillance tool. The use of mobile phone-based tools for surveillance of diseases has expanded in the last decade[32,33]. The uptake and success of these tools is dependent on a number of factors including costs of data transmission, ease of use by the data collectors, and availability of infrastructure such as a good mobile network. In our study, successful use of mobile phones to report disease events can be attributed to a high proportion (> 70%) of mobile phone ownership among study households, a strong mobile network signal (most households receive mobile network signals), and the use of the toll-free option, which takes away the cost of the phone-call from the farmer.

The use of toll-free reporting system alone would possibly miss disease events that farmers view as less serious to warrant reporting. Such cases missed by the toll-free system are picked during the bi-weekly home visits. However, the timely response by the PBASS veterinary team to disease events reported by the farmer, and the offer of free veterinary advice (clinical diagnosis and limited care) for syndromes relevant to the study have been an effective motivator for farmers to report disease events. The combination of a toll-free number and biweekly visits allows analysis of the quality of disease incidence data collected by each pathway, with each providing a quality control check. Over time, we will better assess whether one method is preferable over the other. However, we postulate that with proper feedback offered to the farmers, the use of a toll-free reporting system may be an attractive and cost-effective tool to increase disease surveillance, especially in resource-limited areas.

The platform allows for the study of shocks on household’s health and wealth that may be associated with morbidity and mortality among livestock attributable to infectious diseases. Data on health of livestock through the surveillance system will likely be related to household expenditures on health, health-care seeking following illness in a member of the household, and income and asset changes over the same period of time. The longitudinal nature of data collection provides a chance to relate changes in household assets and consumption with illness and death events in animals. Data will be analysed by syndromes to determine which of the livestock syndromes under study have the greatest economic impacts on households, and the results used to prioritize which interventions will not only reduce livestock disease burden but maximize protection of household assets and livelihoods.

Analysis of data on consumption of animal source foods indicates households that keep cattle and those that keep chickens have an increased likelihood of consuming milk and eggs respectively. While such a correlation might seem intuitive, it is not uncommon that milk and eggs are used for household income to purchase less expensive sources of food with less nutritive value. The consumption of animal source foods is important as it has been found to be positively associated with increased cognitive development and a reduced risk of malnutrition and stunting in children[34–36]. In order to better understand the relationships between livestock ownership and health, and human nutrition, this study is collecting monthly anthropometric data from children under 5 years of age, accompanied by records of nutritional intakes of these same children for a period of at least one year. Linked to the human health data, these outcome variables will be used to investigate the nutritional pathways and determine the impact of accessing animal source foods on disease susceptibility and overall child health, nutritional status, and growth and development.

Our preliminary analysis shows a positive association between the likelihood of spending money on health and the number of chickens owned, and no association with the number of small or large stock owned. Whereas it is plausible to relate health spending to livestock ownership, it is curious why the relationship is significant with poultry alone and not with the other livestock species. To illustrate some of the complexity of household economic decisions that can be examined using our platform, note that the average amount of health spending is relatively small (approximately 5 US dollars a month) and it may be that different livestock species serve different livelihood roles. These spending patterns and associations with specific livestock species may be related to gender roles and livestock ownership. For instance, women are known to have more rights over chicken and sometimes small stock than they have over larger livestock, and are likely to spend significantly higher amounts of their income on families compared to men[37]. There is need for additional studies and analysis to provide information on the intra-household dynamics of ownership, management and marketing of livestock products to draw stronger inferences on relationship between livestock ownership and productivity and human health and nutrition.

Livestock are economically substantial assets. The sale of larger (more valuable) stock may be reserved to cushion against larger household financial needs such as education fees for children, whereas the sale of a chicken may represent a smaller “withdrawal” for the purpose of paying these smaller expenditures. These complexities that drive household spending may be responsible for whether a family gets into or out of poverty and disease[38]. Continuing longitudinal data from longer periods of follow-up will allow for more robust testing of hypotheses on the role different livestock species in meeting specific household needs such as nutrition, cash, education, and socio-cultural needs of livestock keepers.

It is important to note that while animal illness is included in these regressions as an explanatory variable for human illness, these results should not be taken to imply directional causality of livestock health on human health, but instead should be interpreted carefully as qualitative evidence of a (potentially complex) relationship between human and animal health.

In conclusion, the PBASS-PBIDS platform produces a unique opportunity to link human health, animal health and socio-economic data, collected frequently and simultaneously in the same households over the same period of time. These data allow for the investigation, understanding and quantification of the pathways by which human health and welfare are linked to animal health, and provide a platform for testing hypotheses related to “one-health” including scientific enquiries focusing on specific diseases, co-infections and their interactions. While it is tempting to suggest that intervention in animals to control gastrointestinal or respiratory illness may have its most important direct impact on human illness, data on household welfare may indicate that animal health interventions, not necessarily related to a specific disease syndrome, increases household wealth, which, in turn may have the greatest impact on human health. Such data and analyses may have novel contributions to guide health policy and investment in resource constrained areas.

## Supporting Information

S1 Table Summary of the demographic characteristics of households enrolled in the PBASS study. (DOCX)

S2 Table Summary of asset ownership by household. (DOCX)

## References

[1] Staal S, Poole J, Baltenweck I, Mwacharo J. Targeting strategic investment in livestock development as a vehicle for rural livelihoods, Report to Bill and Melinda Gates Foundation. ILRI, Nairobi; 2009.

[2] Randolph TF, Schelling E, Grace D, Nicholson CF, Leroy JL, Cole DC, et al Invited review: Role of livestock in human nutrition and health for poverty reduction in developing countries. J Anim Sci. 2007;85(11):2788–800. 17911229 10.2527/jas.2007-0467

[3] Perry B, Grace D. The impacts of livestock diseases and their control on growth and development processes that are pro-poor. Philos Trans R Soc Lond B Biol Sci. 2009;364(1530):2643–55. 10.1098/rstb.2009.0097 19687035 PMC2865091

[4] Bradford G. Contributions of animal agriculture to meeting global human food demand. Livest Prod Sci. 1999 Jun;59(2–3):95–112.

[5] Sigman M, Neumann C, Baksh M, Bwibo N, McDonald MA. Relationship between nutrition and development in Kenyan toddlers. J Pediatr. 1989 Sep;115(3):357–64. 2769494 10.1016/s0022-3476(89)80832-7

[6] Nicholson CF, Mwangi L, Staal SJ, Thornton PK. Dairy Cow Ownership and Child Nutritional Status in Kenya. 2003 AAEA Annual Meetings. Montréal, Québec, Canada; 2003.

[7] Whaley SE, Sigman M, Neumann C, Bwibo N, Guthrie D, Weiss RE, et al The impact of dietary intervention on the cognitive development of Kenyan school children. J Nutr. 2003 Nov;133(11 Suppl 2):3965S–3971S. 14672297 10.1093/jn/133.11.3965S

[8] Lannotti L, Lesorogol C. Animal milk sustains micronutrient nutrition and child anthropometry among pastoralists in Samburu, Kenya. Am J Phys Anthropol. 2014 Jun 18;00(January). 10.1002/ajpa.22547 24942144

[9] Bhaskaram P. Micronutrient malnutrition, infection, and immunity: an overview. Nutr Rev. 2002 May;60(5 Pt 2):S40–5. 12035857 10.1301/00296640260130722

[10] Hughes S, Kelly P. Interactions of malnutrition and immune impairment, with specific reference to immunity against parasites. Parasite Immunol. 2006 Dec;28(11):577–88. 17042929 10.1111/j.1365-3024.2006.00897.x PMC1636690

[11] Mableson HE, Okello A, Picozzi K, Welburn SC. Neglected zoonotic diseases-the long and winding road to advocacy. PLoS Negl Trop Dis. 2014 Jun;8(6):e2800 10.1371/journal.pntd.0002800 24901769 PMC4046968

[12] Thornton PK, Kruska RL, Henninger N, Kristjanson PM, Reid RS, Atieno F, et al Mapping Poverty and Livestock in the Developing World. Nairobi, Kenya: International Livestock Research Institute; 2002.

[13] Okwi PO, Ndeng’e G, Kristjanson P, Arunga M, Notenbaert A, Omolo A, et al Spatial determinants of poverty in rural Kenya. Proc Natl Acad Sci U S A. 2007 Oct 23;104(43):16769–74. 17942704 10.1073/pnas.0611107104 PMC2040447

[14] Feikin DR, Olack B, Bigogo GM, Audi A, Cosmas L, Aura B, et al The Burden of Common Infectious Disease Syndromes at the Clinic and Household Level from Population-Based Surveillance in Rural and Urban Kenya. Beeson JG, editor. PLoS One. 2011 Jan 18;6(1):e16085 10.1371/journal.pone.0016085 21267459 PMC3022725

[15] Knobel DL, Maina AN, Cutler SJ, Ogola E, Feikin DR, Junghae M, et al Coxiella burnetii in humans, domestic ruminants, and ticks in rural western Kenya. Am J Trop Med Hyg. 2013 Mar;88(3):513–8. 10.4269/ajtmh.12-0169 23382156 PMC3592534

[16] Eshitera EE, Githigia SM, Kitala P, Thomas LF, Fèvre EM, Harrison LJS, et al Prevalence of porcine cysticercosis and associated risk factors in Homa Bay District, Kenya. BMC Vet Res. 2012 Jan;8:234 10.1186/1746-6148-8-234 23217158 PMC3528429

[17] Githigia SM, Willingham AL, Njeruh FM. Palpable lingual cysts, a possible indicator of porcine cysticercosis, in Teso District, Western Kenya. 2007;15(August):206–12.

[18] Kotloff KL, Nataro JP, Blackwelder WC, Nasrin D, Farag TH, Panchalingam S, et al Burden and aetiology of diarrhoeal disease in infants and young children in developing countries (the Global Enteric Multicenter Study, GEMS): a prospective, case-control study. Lancet. 2013 Jul 20;382(9888):209–22. 10.1016/S0140-6736(13)60844-2 23680352

[19] Von Wissmann B, Machila N, Picozzi K, Fèvre EM, deC Bronsvoort BM, Handel IG, et al Factors associated with acquisition of human infective and animal infective trypanosome infections in domestic livestock in Western Kenya. PLoS Negl Trop Dis. 2011 Jan;5(1):e941 10.1371/journal.pntd.0000941 21311575 PMC3022529

[20] Zinsstag J, Schelling E, Wyss K, Mahamat MB. Potential of cooperation between human and animal health to strengthen health systems. Lancet. 2005 Dec 17;366(9503):2142–5. 16360795 10.1016/S0140-6736(05)67731-8

[21] Morse SS, Mazet JA, Woolhouse M, Parrish CR, Carroll D, Karesh WB, et al Prediction and prevention of the next pandemic zoonosis. Lancet. Elsevier Ltd; 2012 Dec;380(9857):1956–65. 10.1016/S0140-6736(12)61684-5 23200504 PMC3712877

[22] Adazu K, Lindblade K a, Rosen DH, Odhiambo F, Ofware P, Kwach J, et al Health and demographic surveillance in rural western Kenya: a platform for evaluating interventions to reduce morbidity and mortality from infectious diseases. Am J Trop Med Hyg. 2005 Dec;73(6):1151–8. 16354829

[23] Odhiambo FO, Laserson KF, Sewe M, Hamel MJ, Feikin DR, Adazu K, et al Profile: the KEMRI/CDC Health and Demographic Surveillance System—Western Kenya. Int J Epidemiol. 2012 Aug;41(4):977–87. 10.1093/ije/dys108 22933646 PMC12083774

[24] Feikin DR, Audi A, Olack B, Bigogo GM, Polyak C, Burke H, et al Evaluation of the optimal recall period for disease symptoms in home-based morbidity surveillance in rural and urban Kenya. Int J Epidemiol. 2010 Apr;39(2):450–8. 10.1093/ije/dyp374 20089695 PMC2846445

[25] R Core Team. R: A Language and Environment for Statistical Computing. Vienna, Austria; {ISBN} 3-900051-07-0, 2014.

[26] Mazet JAK, Clifford DL, Coppolillo PB, Deolalikar AB, Erickson JD, Kazwala RR. A “one health” approach to address emerging zoonoses: the HALI project in Tanzania. PLoS Med. 2009 Dec;6(12):e1000190 10.1371/journal.pmed.1000190 20016689 PMC2784942

[27] Narrod C, Zinsstag J, Tiongco M. A one health framework for estimating the economic costs of zoonotic diseases on society. Ecohealth. 2012 Jun 7;9(2):150–62. 10.1007/s10393-012-0747-9 22395956 PMC3415616

[28] Grace D. The business case for One Health. Onderstepoort J Vet Res. 2014 Jan;81(2):E1–6. 10.4102/ojvr.v81i2.720 25005124

[29] Singer M, Bulled N. Interlocked infections: the health burdens of syndemics of neglected tropical diseases. Ann Anthropol Pract. 2012 Nov 12;36(2):328–45.

[30] Marmot M, Friel S, Bell R, Houweling T a J, Taylor S. Closing the gap in a generation: health equity through action on the social determinants of health. Lancet. 2008 Nov 8;372(9650):1661–9. 10.1016/S0140-6736(08)61690-6 18994664

[31] Argent J, Augsburg B, Rasul I. Livestock asset transfers with and without training: Evidence from Rwanda. J Econ Behav Organ. Elsevier B.V.; 2014 Aug;

[32] Aanensen DM, Huntley DM, Feil EJ, Al-Own F, Spratt BG. EpiCollect: linking smartphones to web applications for epidemiology, ecology and community data collection. PLoS One. 2009 Jan;4(9):e6968 10.1371/journal.pone.0006968 19756138 PMC2735776

[33] Madder M, Walker JG, VAN Rooyen J, Knobel D, Vandamme E, Berkvens D, et al. e-Surveillance in Animal Health: use and evaluation of mobile tools. Parasitology. 2012 Apr 13;1–12. 10.1017/S0031182012000571 22717001

[34] Murphy S, Allen L. Nutritional Importance of Animal Source Foods. J Nutr. 2003;3932–5. 10.1093/jn/133.11.3932S 14672292

[35] Darapheak C, Takano T, Kizuki M, Nakamura K, Seino K. Consumption of animal source foods and dietary diversity reduce stunting in children in Cambodia. Int Arch Med. International Archives of Medicine; 2013 Jul 17;6(1):29. 10.1186/1755-7682-6-29 PMC3720190 23866682

[36] Jin M, Iannotti LL. Livestock production, animal source food intake, and young child growth: The role of gender for ensuring nutrition impacts. Soc Sci Med. Elsevier Ltd; 2014 Mar;105:16–21. 10.1016/j.socscimed.2014.01.001 24606793

[37] Njuki J, Sanginga P. Gender and livestock: Issues, challenges and opportunities Bridging the Gender Gap: Women, Livestock Ownership and Markets in Eastern and Southern Africa. Nairobi, Kenya: International Livestock Research Institute; 2013.

[38] Kristjanson P, Krishna A, Radeny M, Nindo W. Pathways out of Poverty in Western Kenya and the Role of Livestock. Policy. Rome (Italy): FAO; 2004 p. 23. Report No.: 14.

