# Supplementary material for: Linking Human Health and Livestock Health: A “One-Health” Platform for Integrated Analysis of Human Health, Livestock Health, and Economic Welfare in Livestock Dependent Communities
Source: PLoS One. 2015 Mar 23;10(3):e0120761. doi: 10.1371/journal.pone.0120761 (PMC4370696; doi:10.1371/journal.pone.0120761)
Supplement: S1 Table — (DOCX) [file pone.0120761.s001.docx]

Supplementary table S1: Summary of the demographic characteristics of households enrolled in the PBASS study.

| Characteristics | Frequency (%) |
| --- | --- |
| Mean age (SD) | 53.3 (17.1) |
| Gender |  |
| Male | 714 (50.1) |
| Female | 711 (49.9) |
| Education level |  |
| No formal education | 203 (14.5) |
| Primary education | 917 (65.4) |
| Secondary education | 233 (16.6) |
| Tertiary education | 50 (3.6) |
| Primary Occupation |  |
| Employed full-time on farm | 806 (56.6) |
| Employed part time on farm | 128 (9.0) |
| Self-employed off farm | 232 (16.3) |
| Salaried off farm | 76 (5.3) |
| Other | 183 (12.8) |
| Households with Children |  |
| Under 3 years | 476 (33) |
| Under 5 years | 611 (42.3) |
| Under 10 years | 813 (56.3) |
