# Supplementary material for: Linking Human Health and Livestock Health: A “One-Health” Platform for Integrated Analysis of Human Health, Livestock Health, and Economic Welfare in Livestock Dependent Communities
Source: PLoS One. 2015 Mar 23;10(3):e0120761. doi: 10.1371/journal.pone.0120761 (PMC4370696; doi:10.1371/journal.pone.0120761)
Supplement: S2 Table — (DOCX) [file pone.0120761.s002.docx]

Supplementary Table S2: Summary of asset ownership by household

| Assets | Frequency (%) |
| --- | --- |
| Own implements | 1396 (97) |
| Draft implements | 226 (16) |
| Hand implements | 1393 (96) |
| Own bike | 661 (46) |
| Own vehicle | 15 (1) |
| Own radio | 1032 (72) |
| Own tractors | 0 (0) |
| Own phones | 1028 (71) |
| Own TV | 164 (11) |
| Own computer | 7 (0.5) |
| Own electronics | 31 (2) |
